# Supplementary material for: A three dimensional immunolabeling method with peroxidase-fused nanobodies and fluorochromized tyramide-glucose oxidase signal amplification
Source: Commun Biol. 2025 Jun 18;8:903. doi: 10.1038/s42003-025-08317-z (PMC12177075; doi:10.1038/s42003-025-08317-z)
Supplement: Supplementary file 1 — Supplementary Information [file 42003_2025_8317_MOESM1_ESM.pdf]

Supplementary Information

Title:
A Three Dimensional Immunolabeling Method with Peroxidase-fused Nanobodies and Fluorochromized Tyramide-Glucose Oxidase Signal Amplification

Authors:
Kenta Yamauchi, Masato Koike, Hiroyuki Hioki

Supplementary Figure 1. GFP1 POD-nAb immunoreactivity in brain slices processed for tissue permeabilization.

Supplementary Figure 2. RFP6 POD-nAb immunoreactivity in brain slices processed for tissue permeabilization.

Supplementary Figure 3. Specificity of RFP6 POD-nAb/FT-GO 3D-IHC

Supplementary Figure 4. 3D-IHC with an Alexa Fluor 647-conjugated anti-GFP nAb.

Supplementary Figure 5. Reaction time comparison of FT-GO in POD-nAb/FT-GO 3D-IHC.

Supplementary Figure 6. Staining homogeneity of POD-nAb/FT-GO 3D-IHC.

Supplementary Figure 7. The detection of activated microglia with an ITGAM POD-nAb.

Supplementary Figure 8. GFP POD-nAb/FT-GO 3D-IHC in 2-mm-thick brain slices of PV-FGL mice.

Legends for Supplementary Movies.

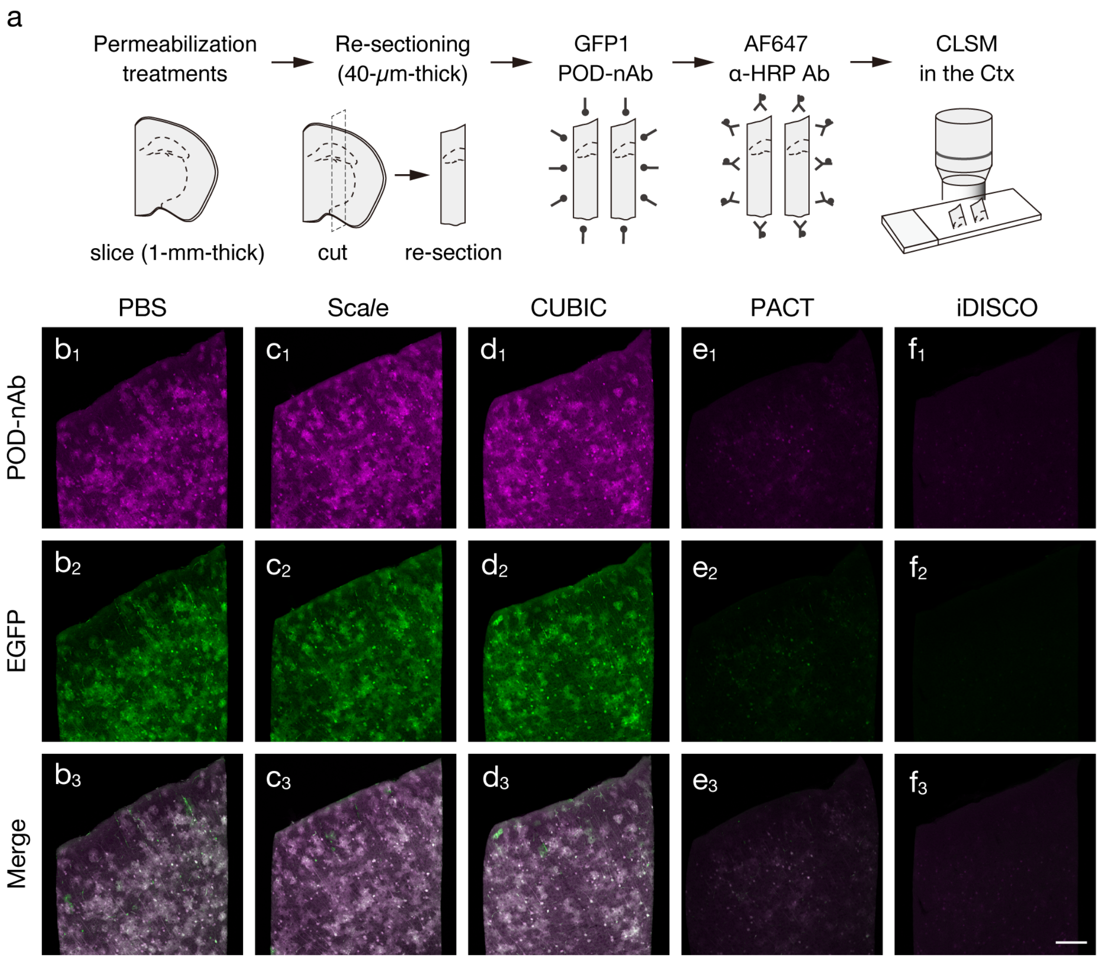

Supplementary Figure 1| GFP1 POD-nAb immunoreactivity in brain slices processed for tissue permeabilization.
a) Schematic diagram of an experimental procedure for testing tissue permeabilization on POD-nAb immunoreactivity. Brain slices are infected with AAV2/PHP.eB CAG-EGFP-WPRE. **b-f**) GFP1 POD-nAb IHC in mouse brain slices processed for PBS (**b**), Sca*l*e (**c**), CUBIC (**d**), PACT (**e**) and iDISCO (**f**) tissue permeabilization (n = 3 animals for each condition). $\mathbf{b_{1,2}}$-$\mathbf{f_{1,2}}$) Representative images of immunoreactivity for GFP1 POD-nAb (magenta, $\mathbf{b_1}$-$\mathbf{f_1}$) and EGFP fluorescence (green, $\mathbf{b_2}$-$\mathbf{f_2}$) in the cerebral cortex. $\mathbf{b_3}$-$\mathbf{f_3}$) Merged images of the GFP1 POD-nAb immunoreactivity and EGFP fluorescence. Images are acquired with the same parameters for comparisons. AF: Alexa Fluor, Ctx: cerebral cortex. Scale bar: 200 μm.

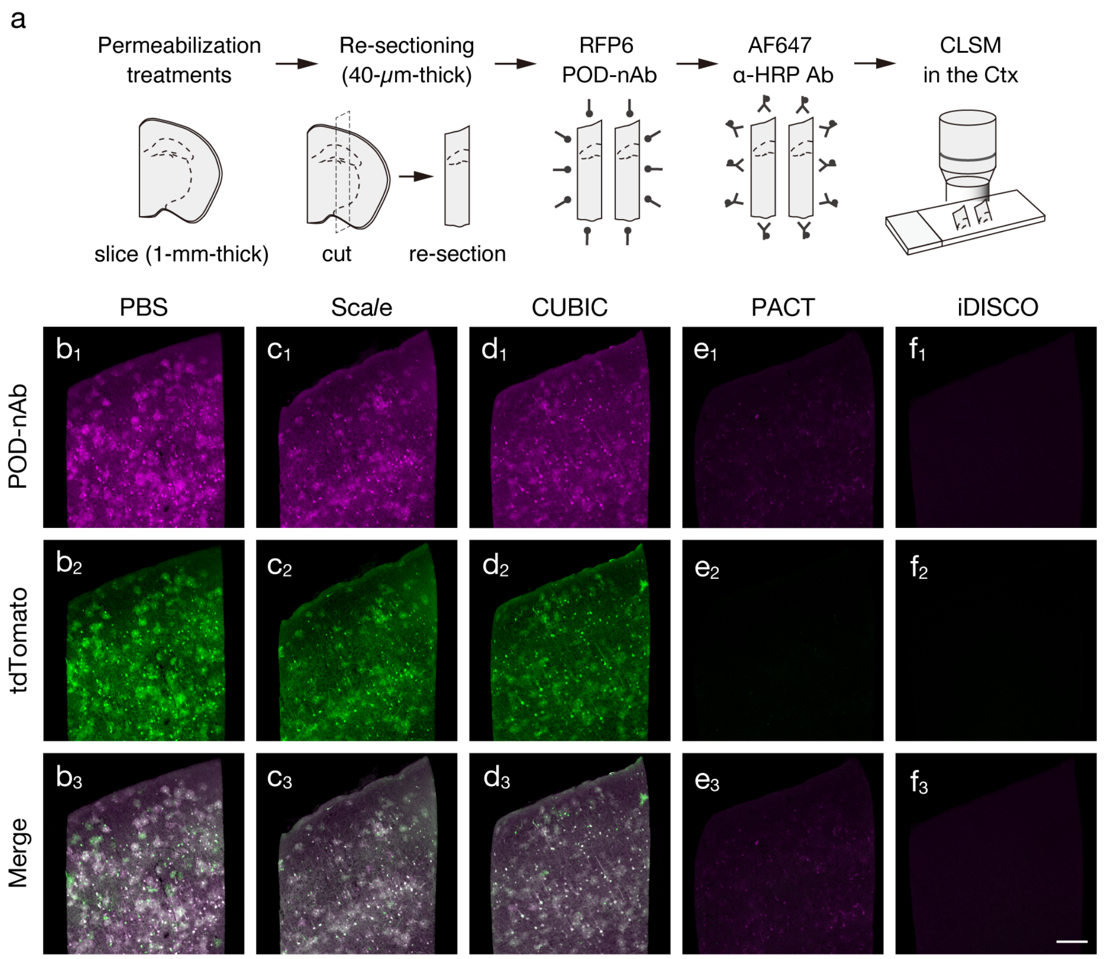

Supplementary Figure 2| RFP6 POD-nAb immunoreactivity in brain slices processed for tissue permeabilization. **a**) Schematic diagram of an experimental procedure for testing tissue permeabilization on POD-nAb immunoreactivity. Brain slices are infected with AAV2/PHP.eB CAG-tdTomato-WPRE. **b-f**) RFP6 POD-nAb IHC in mouse brain slices processed for PBS (**b**), Sca*l*e (**c**), CUBIC (**d**), PACT (**e**) and iDISCO (**f**) tissue permeabilization (n = 3 animals for each condition). $\mathbf{b_{1,2}}$-$\mathbf{f_{1,2}}$) Representative images of immunoreactivity for RFP6 POD-nAb (magenta, $\mathbf{b_1}$-$\mathbf{f_1}$) and tdTomato fluorescence (green, $\mathbf{b_2}$-$\mathbf{f_2}$) in the cerebral cortex. $\mathbf{b_3}$-$\mathbf{f_3}$) Merged images of the RFP6 POD-nAb immunoreactivity and tdTomato fluorescence. Images are acquired with the same parameters for comparisons. AF: Alexa Fluor, Ctx: cerebral cortex. Scale bar: 200 μm.

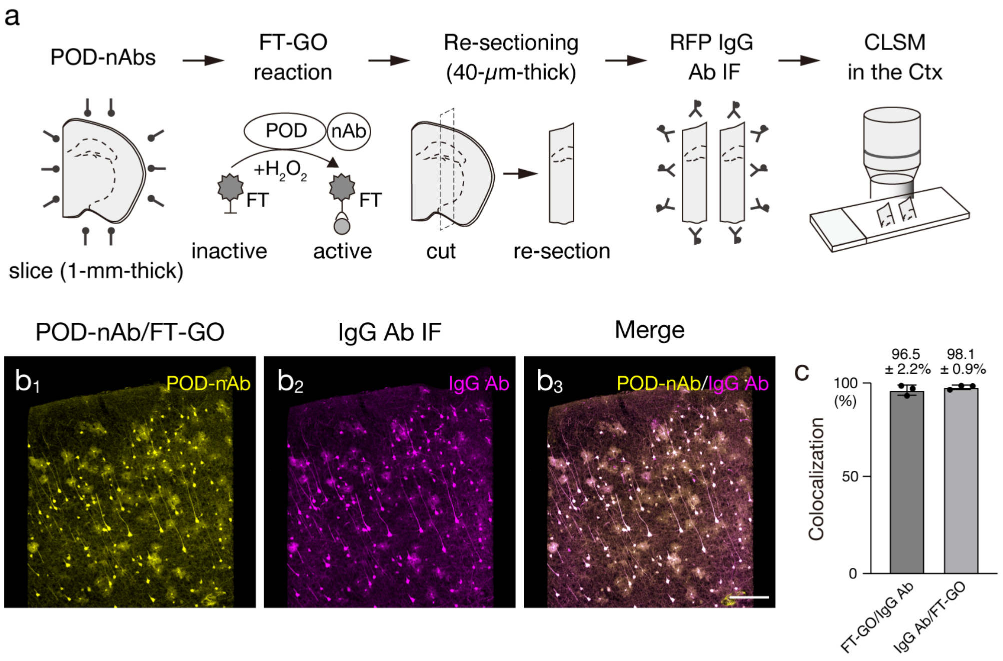

Supplementary Figure 3| Specificity of RFP6 POD-nAb/FT-GO 3D-IHC.

a) Schematic diagram of an experimental procedure for testing specificity of RFP6 POD-nAb/FT-GO 3D-IHC. Re-sections prepared from brain slices subjected to RFP6 POD-nAb/FT-GO 3D-IHC are immunostained with an anti-RFP IgG Ab. The brain slices are infected with AAV2/PHP.eB CAG-tdTomato-WPRE. $\mathbf{b}_{1,2}$) Representative images of RFP6 POD-nAb/FT-GO 3D-IHC (yellow, $\mathbf{b}_1$) and RFP IgG Ab IF (magenta, $\mathbf{b}_2$) in the cerebral cortex. $\mathbf{b}_3$) A merged image of the RFP6 POD-nAb/FT-GO 3D-IHC (yellow) and RFP IgG Ab IF (magenta). **c**) Histograms showing the percentages of FT-GO$^+$ cells in IgG Ab$^+$ cells and IgG Ab$^+$ cells in FT-GO$^+$ cells (n = 460 cells, RFP IF$^+$ cells; n = 477 cells, FT-GO$^+$ cells from 3 animals). Data are represented as means ± SDs. Ctx: cerebral cortex, FT: fluorochromized tyramide, IF: immunofluorescence. Scale bar: 200 μm.

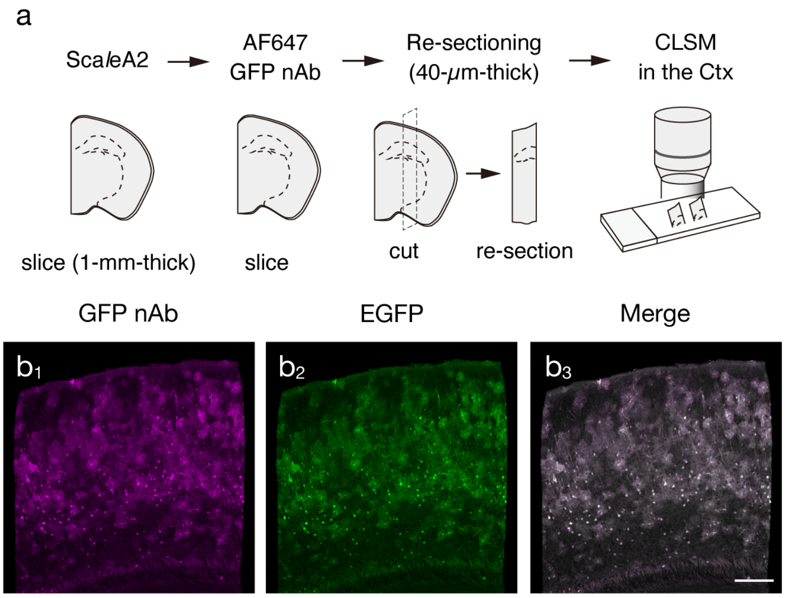

Supplementary Figure 4| 3D-IHC with an Alexa Fluor 647-conjugated anti-GFP nAb.

a) Schematic diagram of 3D-IHC with an Alexa Fluor 647-conjugated anti-GFP nAb and its analysis. Brain slices are infected with AAV2/PHP.eB CAG-EGFP-WPRE. **b**) GFP nAb immunoreactivity in a re-section prepared from a 1-mm-thick mouse brain slice processed for 3D-IHC (n = 3 animals). $\mathbf{b}_{1,2}$) Representative images of immunoreactivity for the GFP nAb (magenta, $\mathbf{b}_1$) and EGFP fluorescence (green, $\mathbf{b}_2$). $\mathbf{b}_3$) A merged image of ($\mathbf{b}_1$) and ($\mathbf{b}_2$). AF: Alexa Fluor. Scale bar: 200 μm.

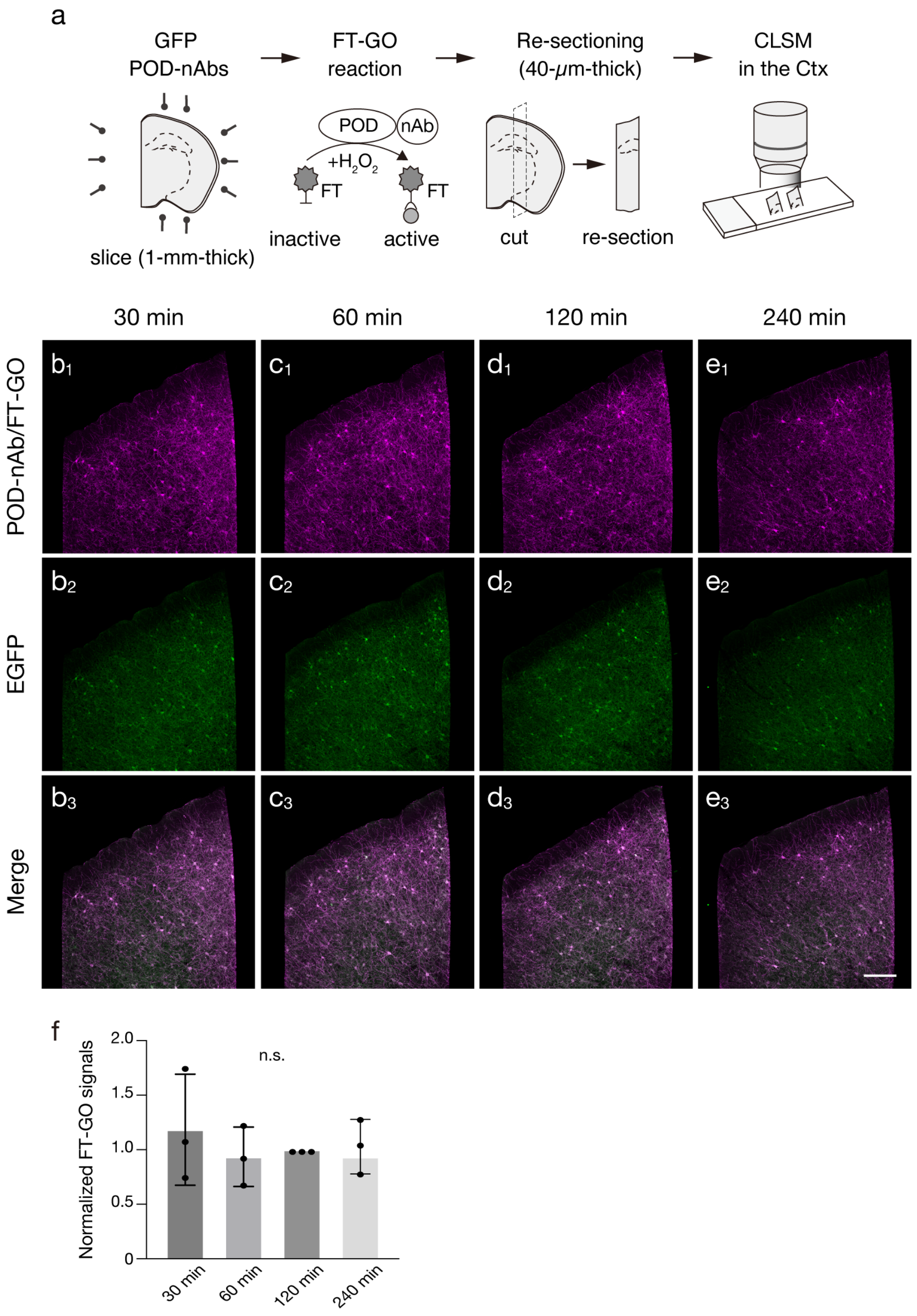

Supplementary Figure 5| Reaction time comparison of FT-GO in POD-nAb/FT-GO 3D-IHC.

a) Schematic diagram of an experimental procedure for testing reaction times of FT-GO. **b-e**) Re-sections prepared from 1-mm-thick brain slices of PV-FGL mice stained by GFP POD-nAb/FT-GO 3D-IHC. Four reaction times 30 (**b**), 60 (**c**), 120 (**d**) or 240 (**e**) min, are tested. $\mathbf{b}_{1,2}$-$\mathbf{e}_{1,2}$) Representative images of FT-GO signals of GFP POD-nAb/FT-GO 3D-IHC (magenta, $\mathbf{b}_1$-$\mathbf{e}_1$) and EGFP fluorescence (green, $\mathbf{b}_2$-$\mathbf{e}_2$) in the cerebral cortex. $\mathbf{b}_3$-$\mathbf{e}_3$) Merged images of the FT-GO signals (magenta) and EGFP fluorescence (green). Images are acquired with the same parameters for comparisons. **f**) Histograms showing signal intensity values of GFP POD-nAb/FT-GO 3D-IHC normalized to those of EGFP fluorescence. These signal intensity values are further normalized by those of 120 min reaction time (n = 3 slices from 4 animals for each condition, H = 1.066, df = 3, P = 0.835, η^2 = 0.097, Kruskal-Wallis test). Data are represented as means ± SDs. Ctx: cerebral cortex, FT: fluorochromized tyramide, n.s.: not significant. Scale bar: 200 μm.

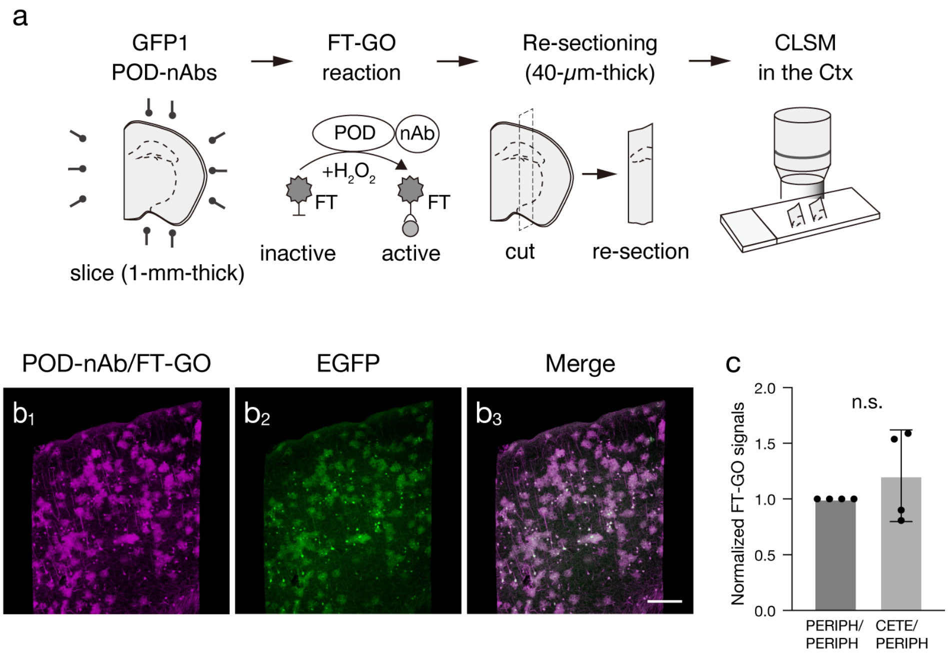

Supplementary Figure 6| Staining homogeneity of POD-nAb/FT-GO 3D-IHC.

a) Schematic diagram of an experimental procedure for testing staining homogeneity of POD-nAb/FT-GO 3D-IHC. **b**) A re-section prepared from a brain slice stained by GFP POD-nAb/FT-GO 3D-IHC. A brain slice of 1-mm thickness infected with AAV2/PHP.eB CAG-EGFP-WPRE is subjected to the 3D-IHC. $\mathbf{b}_{1,2}$) Representative images of FT-GO signals of GFP POD-nAb/FT-GO 3D-IHC (magenta, $\mathbf{b}_1$) and EGFP fluorescence (green, $\mathbf{b}_2$) in the cerebral cortex. $\mathbf{b}_3$) A merged image of the FT-GO signals (magenta) and EGFP fluorescence (green). **c**) Histograms showing signal intensity values of GFP POD-nAb/FT-GO 3D-IHC normalized to those of EGFP fluorescence in the periphery (≤ 100 µm from the surface) and the center (≥ 400 µm from the surface) of brain slices. These signal intensity values are further normalized by those in the periphery of brain slices (n = 4 animals, $t = 1.015$, $df = 3$, $P = 0.3848$, $r = 0.5056$, Paired t test). Data are represented as means ± SDs. CETE: center, Ctx: cerebral cortex, FT: fluorochromized tyramide. n.s.: not significant, PERIPH: periphery. Scale bar: 200 µm.

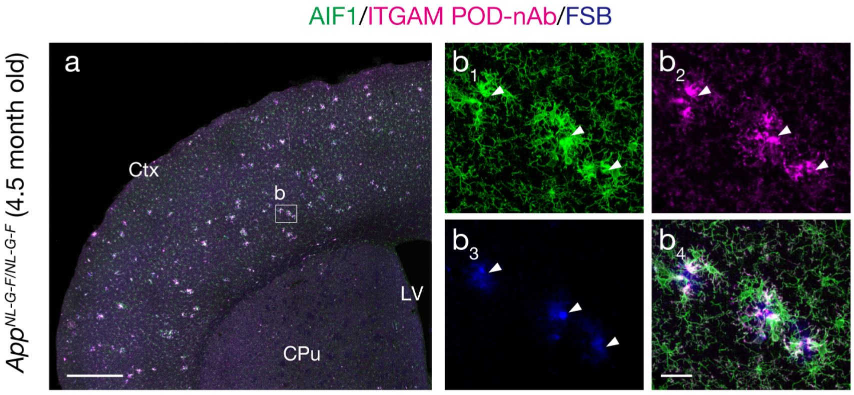

Supplementary Figure 7| The detection of activated microglia with an ITGAM POD-nAb.

a) An immunostaining with an AIF1 IgG Ab (green) and an ITGAM POD-nAb (magenta) in an *App*$^{NL\text{-}G\text{-}F/NL\text{-}G\text{-}F}$ brain section (n = 3 animals). The section is also fluorescently labeled with FSB (blue) for Aß plaque detection. **b**) A higher magnification image in a rectangle in (**a**). $\mathbf{b}_{1\text{-}3}$) Representative images of AIF1 IgG Ab immunoreactivity ($\mathbf{b}_1$), ITGAM POD-nAb immunoreactivity ($\mathbf{b}_2$) and FSB labeling ($\mathbf{b}_3$). $\mathbf{b}_4$) A merged image of ($\mathbf{b}_1$), ($\mathbf{b}_2$) and ($\mathbf{b}_3$). Arrowheads indicate the positions of Aß plaques labeled with FSB. CPu: caudate-putamen, Ctx: cerabral cortex, LV: lateral ventricle. Scale bar: 500 µm in (**a**) and 30 µm in (**b**).

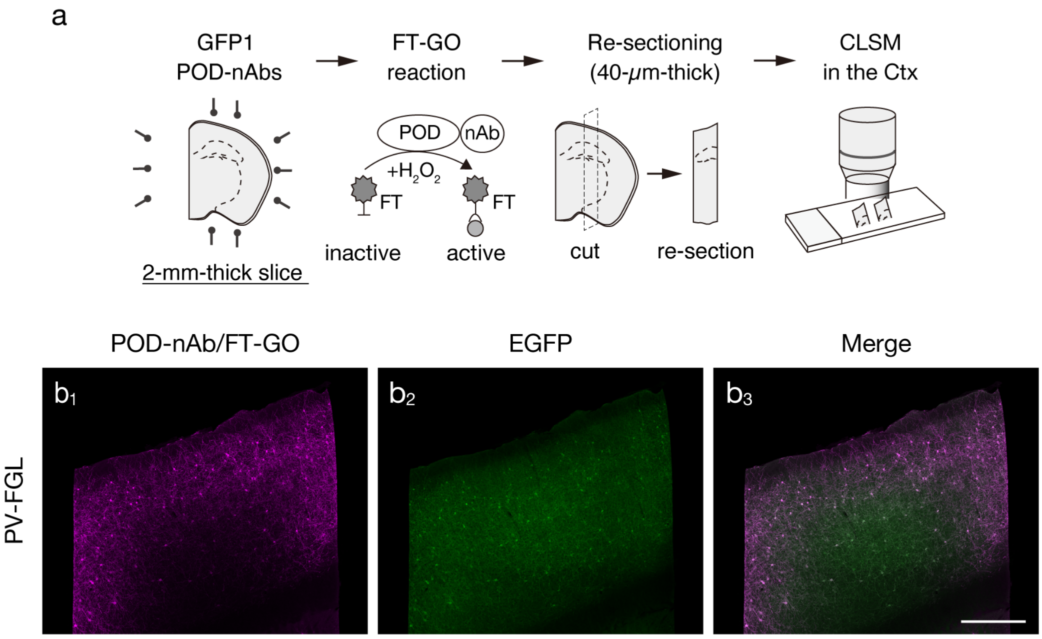

Supplementary Figure 8| GFP POD-nAb/FT-GO 3D-IHC in 2-mm-thick brain slices of PV-FGL mice.

a) Schematic diagram of a experimental procedure for testing signal penetration of POD-nAb/FT-GO 3D-IHC. Note that brain slices of 2-mm thickness of PV-FGL mice are subjected to POD-nAb/FT-GO 3D-IHC (n = 4 slices from 4 animals). **b**) A re-section prepared from a 2-mm-thick brain slice stained by GFP POD-nAb/FT-GO 3D-IHC. $\mathbf{b}_{1,2}$) Representative images of FT-GO signals of GFP POD-nAb/FT-GO 3D-IHC (magenta, $\mathbf{b}_1$) and EGFP fluorescence (green, $\mathbf{b}_2$) in the cerebral cortex. $\mathbf{b}_3$) A merged image of the FT-GO signals (magenta) and EGFP fluorescence (green). Ctx: cerebral cortex, FT: fluorochromized tyramide. Scale bar: 500 μm.

Legends for Supplementary Movies.

Supplementary Movie 1. Serial xy images of a 1-mm-thick mouse brain slice stained by GFP POD-nAb/FT-GO 3D-IHC.

A 1-mm-thick mouse brain slice infected with AAV2/PHP.eB CAG-EGFP-WPRE is stained by GFP POD-nAb/FT-GO 3D-IHC (yellow). CF647 tyramide is used for color development. Following 3D-IHC, the brain slice is cleared with ScaleS4 solution and subjected to CLSM. Images are taken in the cerebral cortex at an interval 1.67 μm from the surface to the depth of 1 mm (n = 3 animals). Scale bar: 25 μm.

Supplementary Movie 2. Serial xy images of ITGAM and GFP double POD-nAb/FT-GO 3D-IHC in a PV-FGL mouse.

Serial xy images of Figure 8d. ITGAM (green) is a maker for microglia. EGFP is expressed by the somatodendritic portion of PV-positive cortical interneurons and labeled by GFP POD-nAb/FT-GO 3D-IHC (magenta). Scale bar: 25 μm.

Supplementary Movie 3. Multiplexed imaging of nucleic acids and POD-nAb/FT-GO 3D-IHC in a 1-mm-thick brain slice.

Serial xy images of the cerebral cortex of a PV-FGL mouse stained by propidium iodide (PI, magenta) and GFP POD-nAb/FT-GO 3D-IHC (cyan). EGFP is expressed by the somatodendritic portion of PV-positive cortical interneurons. CF640R tyramide is used for color development. Following multiplexed labeling, the brain slice is cleared with ScaleS4 solution and subjected to CLSM. Images are taken in the cerebral cortex at an interval 2.5 μm from the surface to the depth of 1 mm (n = 3 animals). Scale bar: 50 μm.

Supplementary Movie 4. Multiplexed imaging of EGFP fluorescence and POD-nAb/FT-GO 3D-IHC in a 1-mm-thick brain slice.

Serial xy images of multiplexed labeling of EGFP fluorescence (magenta) and ITGAM POD-nAb/FT-GO 3D-IHC (yellow) in a 1-mm-thick mouse brain slice infected with AAV2/PHP.eB CAG-EGFP-WPRE. CF640R tyramide is used for color development. Following 3D-IHC, the brain slice is cleared with ScaleS4 solution and subjected to CLSM. Images are taken in the cerebral cortex at an interval 2.5 μm from the surface to the depth of 1 mm (n = 3 animals). Scale bar: 50 μm.
