# Supplementary material for: A three dimensional immunolabeling method with peroxidase-fused nanobodies and fluorochromized tyramide-glucose oxidase signal amplification
Source: Commun Biol. 2025 Jun 18;8:903. doi: 10.1038/s42003-025-08317-z (PMC12177075; doi:10.1038/s42003-025-08317-z)
Supplement: Supplementary file 2 — Description of Additional Supplementary Files [file 42003_2025_8317_MOESM2_ESM.docx]

Description of Additional Supplementary Files

**File name:** Supplementary Movie 1

**Description:** Serial xy images of a 1-mm-thick mouse brain slice stained by GFP POD-nAb/FT-GO 3D-IHC.

**File name:** Supplementary Movie 2

**Description:** Serial xy images of ITGAM and GFP double POD-nAb/FT-GO 3D-IHC in a PV-FGL mouse.

**File name:** Supplementary Movie 3

**Description:** Multiplexed imaging of nucleic acids and POD-nAb/FT-GO 3D-IHC in a 1-mm-thick brain slice.

**File name:** Supplementary Movie 4

**Description:** Multiplexed imaging of EGFP fluorescence and POD-nAb/FT-GO 3D-IHC in a 1- mm-thick brain slice. File name: Supplementary Data 1 Description: The source data behind the graphs in the paper.

**File name:** Supplementary Data 1

**Description:** The source data behind the graphs in the paper.
