# Supplementary material for: A three dimensional immunolabeling method with peroxidase-fused nanobodies and fluorochromized tyramide-glucose oxidase signal amplification
Source: Commun Biol. 2025 Jun 18;8:903. doi: 10.1038/s42003-025-08317-z (PMC12177075; doi:10.1038/s42003-025-08317-z)
Supplement: Supplementary file 8 — Reporting summary [file 42003_2025_8317_MOESM8_ESM.pdf]

nature portfolio

Corresponding author(s): Yamauchi Kenta

Last updated by author(s): May 13, 2025

Reporting Summary

Nature Portfolio wishes to improve the reproducibility of the work that we publish. This form provides structure for consistency and transparency in reporting. For further information on Nature Portfolio policies, see our Editorial Policies and the Editorial Policy Checklist.

Statistics

For all statistical analyses, confirm that the following items are present in the figure legend, table legend, main text, or Methods section.

n/a	Confirmed	
☐	☒	The exact sample size (*n*) for each experimental group/condition, given as a discrete number and unit of measurement
☐	☒	A statement on whether measurements were taken from distinct samples or whether the same sample was measured repeatedly
☐	☒	The statistical test(s) used AND whether they are one- or two-sided<br>*Only common tests should be described solely by name; describe more complex techniques in the Methods section.*
☐	☒	A description of all covariates tested
☐	☒	A description of any assumptions or corrections, such as tests of normality and adjustment for multiple comparisons
☐	☒	A full description of the statistical parameters including central tendency (e.g. means) or other basic estimates (e.g. regression coefficient) AND variation (e.g. standard deviation) or associated estimates of uncertainty (e.g. confidence intervals)
☐	☒	For null hypothesis testing, the test statistic (e.g. *F*, *t*, *r*) with confidence intervals, effect sizes, degrees of freedom and *P* value noted<br>*Give P values as exact values whenever suitable.*
☒	☐	For Bayesian analysis, information on the choice of priors and Markov chain Monte Carlo settings
☒	☐	For hierarchical and complex designs, identification of the appropriate level for tests and full reporting of outcomes
☒	☐	Estimates of effect sizes (e.g. Cohen's *d*, Pearson's *r*), indicating how they were calculated

Our web collection on statistics for biologists contains articles on many of the points above.

Software and code

Policy information about availability of computer code

Data collection	No open source or custom code was used to collect the data in this study.
Data analysis	Leica Application Suite X software (LAS X, ver. 3.5.5.19976, Leica Microsystems) was used for tiling, stitching and creating maximum intensity projection images. Three dimensional rendering images were created using Imaris (ver. 9.9.1, Oxford Instruments) or LAS X software. The global brightness and contrast of the images were adjusted with ImageJ software (ver. 1.53, National Institutes of Health). Quantification of signal intensity was performed with Fiji software (ver. 2.16.0/1.54p, National Institutes of Health). Statistical analyses was performed using GraphPad Prism 9 (Version 9.4.1 (458), GraphPad Software).

For manuscripts utilizing custom algorithms or software that are central to the research but not yet described in published literature, software must be made available to editors and reviewers. We strongly encourage code deposition in a community repository (e.g. GitHub). See the Nature Portfolio guidelines for submitting code & software for further information.

Data

Policy information about availability of data

All manuscripts must include a data availability statement. This statement should provide the following information, where applicable:

- Accession codes, unique identifiers, or web links for publicly available datasets
- A description of any restrictions on data availability
- For clinical datasets or third party data, please ensure that the statement adheres to our policy

The datasets generated during and/or analyzed during the current study and all biological materials reported in this article are available from the corresponding authors (or other sources, as applicable) on reasonable request. Source data can be obtained from Supplementary Data 1

Research involving human participants, their data, or biological material

Policy information about studies with human participants or human data. See also policy information about sex, gender (identity/presentation), and sexual orientation and race, ethnicity and racism.

Reporting on sex and gender	Not applicable.
Reporting on race, ethnicity, or other socially relevant groupings	Not applicable.
Population characteristics	Not applicable.
Recruitment	Not applicable.
Ethics oversight	Not applicable.

Note that full information on the approval of the study protocol must also be provided in the manuscript.

Field-specific reporting

Please select the one below that is the best fit for your research. If you are not sure, read the appropriate sections before making your selection.

☒ Life sciences ☐ Behavioural & social sciences ☐ Ecological, evolutionary & environmental sciences

For a reference copy of the document with all sections, see nature.com/documents/nr-reporting-summary-flat.pdf

Life sciences study design

All studies must disclose on these points even when the disclosure is negative.

Sample size	Sample sizes were chosen on the basis of similar experiments that were previously published (Furuta et al., iScience 2021; Yamauchi et al., Sci Rep 2022). Statistical methods were not used to predetermine and/or re-calculate sample sizes.
Data exclusions	No data was excluded.
Replication	All findings were confirmed with multiple biological replicates from three or more than three animals as detailed in the figure legends, and the representative results are shown.
Randomization	Samples were randomly allocated to experimental groups. Animals from different cages but within the same experimental group were selected to assure randomization.
Blinding	The investigators were not strictly blinded in this study. However, quantification was performed by those who were blind to the identity of experimental groups.

Reporting for specific materials, systems and methods

We require information from authors about some types of materials, experimental systems and methods used in many studies. Here, indicate whether each material, system or method listed is relevant to your study. If you are not sure if a list item applies to your research, read the appropriate section before selecting a response.

Materials & experimental systems

n/a	Involved in the study
☐	☒ Antibodies
☐	☒ Eukaryotic cell lines
☒	☐ Palaeontology and archaeology
☐	☒ Animals and other organisms
☒	☐ Clinical data
☒	☐ Dual use research of concern
☒	☐ Plants

Methods

n/a	Involved in the study
☒	☐ ChIP-seq
☒	☐ Flow cytometry
☒	☐ MRI-based neuroimaging

Antibodies

Antibodies used	Antibodies (Abs) used in this study are described in Methods.<br>Primary Abs used were; guinea pig polyclonal anti-DsRed Ab (DsRed-GP-Af360, Frontier Institute, RRID: AB_2571648), rabbit polyclonal anti-GFP Ab (A-11122, Thermo Fisher Scientific, RRID: AB_221569), rat monoclonal anti-GFP Ab (04404-26, Nacalai Tesque, RRID: AB_2313652), chicken polyclonal anti-Green Fluorescent Protein Ab (GFP-1020, Aves, RRID: AB_10000240), Alexa Fluor 647-conjugated anti-GFP nanobody (Cheomotek, gb2AF647, RRID: AB_2827575), Alexa Fluor 647-conjugated goat anti-HRP Ab (123-605-021, Jackson Immuno Research, RRID: AB_2338967), goat polyclonal anti-Iba1 Ab (011-27991, FUJIFILM Wako Pure Chemical Corporation, RRID:AB_2935833), mouse monoclonal anti-RFP Ab (409 011, Synaptic Systems, RRID: AB_2800533), and goat polyclonal anti-tdTomato Ab (AB8181-200, SICGEN, RRID: AB_2722750).<br>Secondary Abs used were; Alexa Fluor 647-conjugated goat anti-chicken IgY (A-21449, Thermo Fisher Scientific, RRID: AB_2535866), Alexa Fluor 647-conjugated goat anti-guinea pig IgG (A-21450, Thermo Fisher Scientific, RRID: AB_2735091), Alexa Fluor 647-conjugated donkey anti-goat IgG (A-21447, Thermo Fisher Scientific, RRID: AB_2535864), POD-conjugated F(ab')2 fragment donkey anti-goat IgG (705-036-147, Jackson Immuno Research, RRID:AB_2340392), Alexa Fluor 647-conjugated donkey anti-mouse IgG (A-31571, Thermo Fisher Scientific, RRID: AB_162542), Alexa Fluor 647-conjugated goat anti-rabbit IgG (A-21245, Thermo Fisher Scientific, RRID: AB_2535813) and Alexa Fluor 647-conjugated goat anti-rat IgG (A-21247, Thermo Fisher Scientific, RRID: AB_141778).<br>POD-nanobody used were; GFP POD-nAb1, ITGAM POD-nAb, RFP POD-nAb6.
Validation	All the commercially available antibodies were validated for the species (mouse) and application (immunohistochemistry) by the corresponding manufacturer.<br>GFP POD-nAb1 and RFP POD-nAb6 were validated in Yamagata et al., 2018 (doi: 10.1073/pnas.1722491115). The nanobody used in the ITGAM POD-nAb was validated in Rashidian et al., 2016 (doi: 10.1073/pnas.1502609112).

Eukaryotic cell lines

Policy information about cell lines and Sex and Gender in Research

Cell line source(s)	293T cells used in this study were obtained from RIKEN BioResource Research Center (RCB2202).
Authentication	The 293T cell line was authenticated by RIKEN BioResource Research Center.
Mycoplasma contamination	No mycoplasma contamination was detected in the 293T cell line.
Commonly misidentified lines (See ICLAC register)	No commonly misidentified lines were used in this study.

Animals and other research organisms

Policy information about studies involving animals; ARRIVE guidelines recommended for reporting animal research, and Sex and Gender in Research

Laboratory animals	Mouse line: C57BL/6J, 8-16 weeks old.<br>Mouse line: AppNL-G-F (RBRC06344, RIKEN BioResource Research Center; Saito et al., 2014; doi: 10.1038/nn.3697.), 4.5- and 6-month-old.<br>Mouse line: Parvalbumin (PV)/myristoylationEGFP-low-density lipoprotein receptor C-terminal BAC transgenic mice (PV-FGL mice; Kameda et al., 2012, doi: 10.1111/j.1460-9568.2012.08027.x.), 8-16 weeks old.<br>AppNL-G-F and PV-FGL mice were maintained in C57BL/6J background.
Wild animals	No wild animals were used in this study.
Reporting on sex	Randomized male and female mice were used in this study. We did not find significant differences between the genders.
Field-collected samples	Field-collected samples were not used in this study.
Ethics oversight	All animal experiments were approved by the Institutional Animal Care and Use Committees of Juntendo University (Approval No.

Ethics oversight	2021245 and 2021246). All animal procedures were conducted in compliance with ARRIVE (Animal Research: Reporting In Vivo Experiments) guidelines.

Note that full information on the approval of the study protocol must also be provided in the manuscript.

Plants

Seed stocks	Not applicable.
Novel plant genotypes	Not applicable.
Authentication	Not applicable.
